# TGFΒ-induced transcription in cancer

**DOI:** 10.1016/j.semcancer.2016.08.009

**Published:** 2017-02

**Authors:** Gaia Cantelli, Eva Crosas-Molist, Mirella Georgouli, Victoria Sanz-Moreno

**Affiliations:** Tumour Plasticity Laboratory, Randall Division of Cell and Molecular Biophysics, New Hunt’s House, Guy’s Campus, King’s College London, London SE1 1UL, UK

**Keywords:** TGFβ, Cancer, Metastasis, Transcription, EMT, Immunosurveillance

## Abstract

The Transforming Growth Factor-beta (TGFβ) pathway mediates a broad spectrum of cellular processes and is involved in several diseases, including cancer. TGFβ has a dual role in tumours, acting as a tumour suppressor in the early phase of tumorigenesis and as a tumour promoter in more advanced stages. In this review, we discuss the effects of TGFβ-driven transcription on all stages of tumour progression, with special focus on lung cancer. Since some TGFβ target genes are specifically involved in promoting metastasis, we speculate that these genes might be good targets to block tumour progression without compromising the tumour suppressor effects of the TGFβ pathway.

## 1 Introduction

The TGFβ signalling pathway mediates cell proliferation, apoptosis, differentiation, extracellular matrix (ECM) production, cytokine secretion and motility in cancer cells, thus playing a key role in tumour progression [1], [2], [3]. TGFβ ligands such as TGFβ1, TGFβ2 and TGFβ3 belong to the TGFβ superfamily, which also includes other growth factors such as bone morphogenic proteins (BMPs), growth and differentiation factors (GDFs), activins and the anti-mullerian hormone (AMH) [1].

**Fig. 1:**
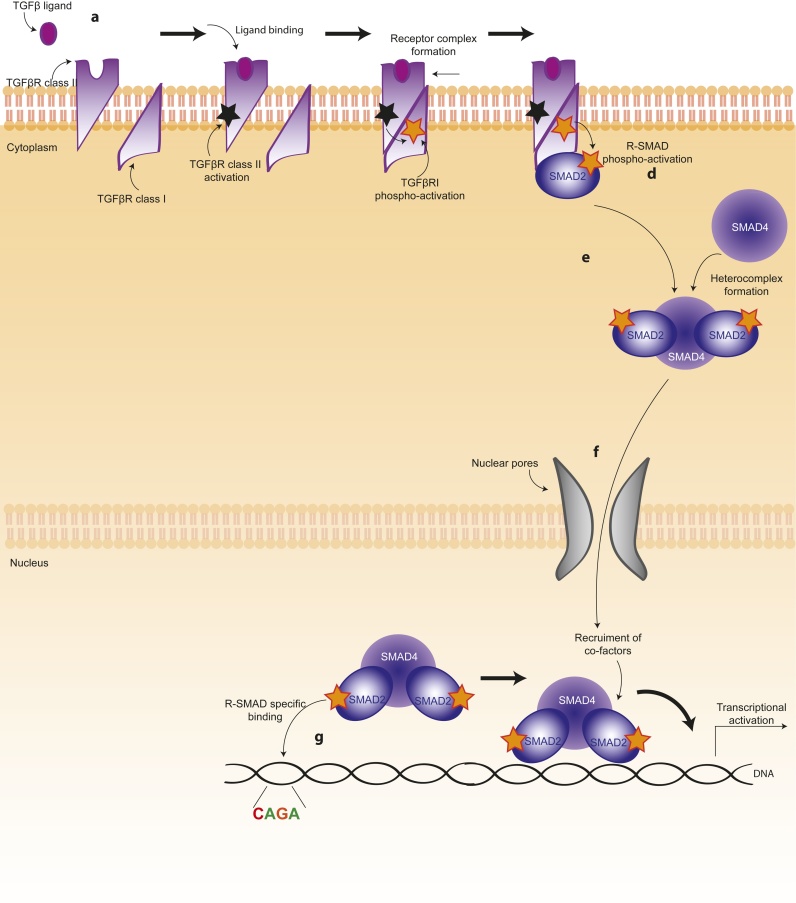
Canonical TGFβ signalling. Diagram summarising canonical TGFβ signalling. TGFβ ligand binding leads to receptor activation, which in turn leads to phospho-activation of R-SMADs. Active R-SMADs bind to SMAD4 to form a heterotrimer that localises to the nucleus, where it drives transcription with the help of several cofactors.

TGFβ ligand binding results in the formation of a hetero-tetrameric complex of type I and type II serine/threonine kinase receptors, where the constitutively active type II receptor phosphorylates and activates the type I receptor. Among the different types of type I and type II receptors, TGFβ preferentially signals through Activin receptor-like kinase 5 (ALK5) type I receptor and the TGFβ type II receptor [4], [5]. Once activated, type I TGFβ receptors phosphorylate members of the R-SMAD family, typically SMAD2 and SMAD3. Phosphorylated R-SMADs associate with SMAD4 to form hetero-trimers. Subsequently, they translocate to the nucleus where, in collaboration with other transcription factors, they regulate transcription of several target genes [6], [7] (Fig. 1). TGFβ-driven transcription is fine-tuned by adaptors, co-activators and co-factors, which are cell- and context-specific, explaining the variety of biological responses elicited by TGFβ stimulation [8]. TGFβ has also been shown to signal independently of SMADs by directly activating RhoA GTPase [9], [10] or alternative signalling pathways [11], [12], [13]. In this review, we will first discuss the role of TGFβ in lung cancer, and then we will expand to other epithelial cancers such as hepatocellular carcinoma (HCC), breast cancer and prostate cancer, and two aggressive non-epithelial cancers in which TGFβ plays an important role, glioblastoma and melanoma.

Lung cancer is one of the leading causes of cancer-related mortality worldwide. There are two main types of lung cancer, small-cell lung cancer (SCLC) and non-small cell lung cancer (NSCLC), the latter being the most common. Because of the asymptomatic course of the disease, most cases are diagnosed at advanced stages, when surgery is no longer an option. Despite the recent advances in lung cancer research, the 5-year survival rate among NSCLC patients remains around 15% [14]. Therefore, a deeper understanding of the molecular mechanisms underlying lung cancer development and progression is needed to develop more effective therapeutic options.

## 2 TGFβ signalling in early stages of cancer development

### 2.1 The TGFβ paradox

TGFβ plays contrasting roles in cancer, acting as a tumour suppressor during the first stages of tumorigenesis and as a tumour promoter during advanced stages of progression [15], [16], [17]. This apparent paradox can be explained by the fact that while some tumours develop TGFβ-inactivating mutations and progress in a TGFβ-independent manner [18], others accumulate mutations in tumour suppressor genes that operate downstream of TGFβ signalling. Cancer cells that acquire these mutations gain a great advantage over their non-mutated counterparts, as they can exploit the wide range of pro-tumorigenic effectors downstream of TGFβ stimulation [16].

For instance, lung cancer cells have been shown to epigenetically silence the TGFβ co-receptor Endoglin in order to exploit the pro-invasive and pro-metastatic effects of TGFβ [19]. Moreover, p53 suppresses the tumour-suppressive functions of TGFβ and promotes its pro-metastatic role in lung cancer by regulating specific sets of TGFβ regulated genes [20]. Similarly, HCC cells epigenetically downregulate TGFβ target gene HEYL, which is thought to suppress tumorigenesis by promoting p53-mediated apoptosis [21]. TGFβ is also a well-established tumour suppressor in the early stages of breast cancer progression [22], [23], [24]. However, TGFβ shifts to a pro-metastatic role at later stages: this switch has been shown to be mediated by the Src regulator PEAK1 [25], highlighting importance of signalling pathway crosstalk during cancer progression.

### 2.2 TGFβ signalling in angiogenesis

Many tumours are able to induce new blood vessel formation, in a process known as angiogenesis (Fig. 3). Angiogenesis allows oxygen and nutrients to reach the inner, less perfused regions of solid tumours [26]. TGFβ secreted by stromal cells as well as by cancer cells themselves has been shown to promote angiogenesis [27], [28]. For instance, TGFβ-driven transcription has been shown to induce angiogenic factors such as VEGF and CTGF in lung cancer and in HCC [29], [30], [31]. Moreover, in prostate cancer inhibition of TGFβ-driven transcription by apigenin decreases VEGF production and overall impaired progression [32]. VEGF expression is similarly controlled by TGFβ-driven transcription in glioblastoma [33], [34]. Glioblastoma-secreted TGFβ also increases expression of insulin-like growth factor-binding protein 7 (IGFBP7) in endothelial cells, promoting angiogenesis [35]. Conversely, endothelial cells stimulate TGFβ signalling in glioblastoma cells, promoting cell migration [36]. Furthermore, in melanoma TGFβ signalling leads to IL-8 secretion, which also supports angiogenesis and capillary formation [37].

### 2.3 TGFβ signalling and cancer-associated fibroblasts

Cancer cells have a profound impact on their microenvironment by promoting the expression and secretion of components of the ECM, matrix metalloproteases (MMPs) and cytokines [1], [5], [38]. Cancer-associated fibroblasts (CAFs) are one of the most important stromal cells in the tumour microenvironment. Indeed, different cell types can become CAFs in response to signals from cancer cells, such as TGFβ [39]. CAFs can promote EMT, both by secreting molecules directed to cancer cells and by remodelling the tumour microenvironment through the secretion of MMPs and helping local invasion [40], [41]. In particular, epithelial cancer cells have been shown to induce the production of MMP9 by stromal fibroblasts, leading to the remodelling of the ECM and TGFβ-driven cancer progression [42], [43]. Moreover, TGFβ from cancer cells induces the expression of MMP1 and fibronectin (FN1) in CAFs [44], [45]. TGFβ also allows for CAFs to acquire pro-invasive qualities. For instance, TGFβ allows CAFs to form functional filopodia and consequently to invade the tumour microenvironment, gaining proximity with cancer cells [46]. Similarly, TGFβ increases actomyosin contractility in fibroblasts by promoting LIF expression [47]. LIF subsequently promotes a pro-invasive phenotype in CAFs by epigenetically activating JAK/STAT signalling, resulting in ECM remodelling and formation of tracks that invading cancer cells follow into the local microenvironment [48], [49]. Finally, CAFs reaching proximity with cancer cells allow them to carry out pro-tumorigenic functions, such as supporting inflammation [50], angiogenesis [51] and tumour initiation [52]. CAFs can also be recruited at secondary tumour sites, where they support metastasis formation [53]. For instance, metastatic breast cancer cells induce CAFs to produce POSTN by secreting TGFβ, thus promoting lung colonisation [54].

In summary, as well as being an established driver of cell motility and local invasion in both epithelial and non-epithelial cells, TGFβ signalling supports cancer-associated phenotypes in fibroblasts. In turn, this promotes EMT, enhancing local invasion and thus promoting tumour progression.

## 3 TGFβ signalling and immune response in cancer

**Fig. 2:**
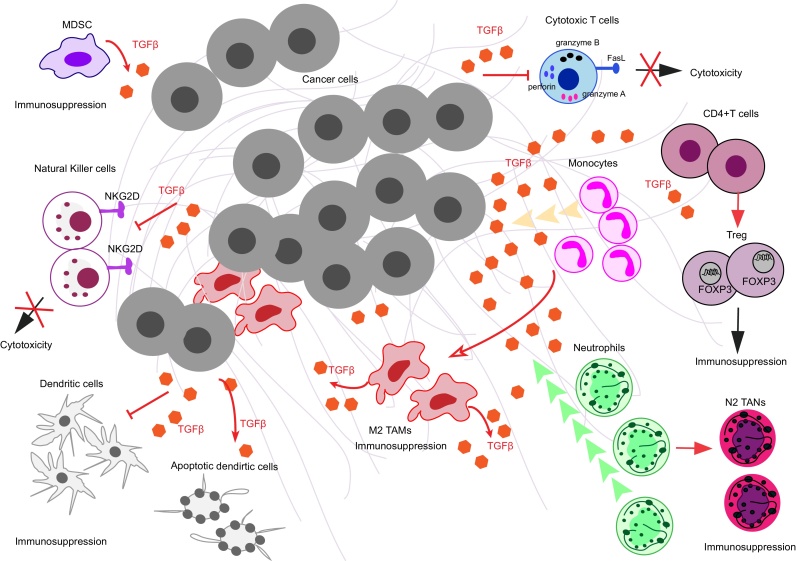
TGFβ signalling effects on immune cells. Diagram showing how TGFβ signalling affects immune cell compartments in the tumour microenvironment. TGFβ can induce monocyte recruitment and then further differentiate and polarize them into M2 tumour-associated macrophages (TAMs). These M2 TAMs can in turn secrete TGFβ supporting tumour promotion. TGFβ can also stimulate neutrophil chemoattraction and then induce a tumour-promoting type of those granulocytes which is called N2 tumour-associated neutrophils (TANs). TGFβ signalling can inhibit effector functions of dendritic cells (DC) or induce their apoptosis. TGFβ inhibits the cytotoxic function of natural killer cells (NK) by down-regulating the NK-specific receptor, NKG2D. Moreover, TGFβ represses cytotoxic gene expression, namely granzyme A, granzyme B, perforin, IFN-γ and FasL in cytotoxic T cells (CTLs). TGFβ can also act on T helper cell differentiation. It induces FOXP3 expression in induced T regulatory cells (Treg) and supports their phenotype and suppressive functions. In addition to tumour-derived TGFβ, myeloid-derived suppressor cells (MDSC) can secrete TGFβ as well. This helps tumour cells evade immune surveillance and sustain tumour progression.

Cancer progression is dependent on escaping immunosurveillance. TGFβ has been shown to maintain immune tolerance and to support tumour-promoting immune cell functions [55], [56], which are key to tumour progression (Fig. 2). TGFβ also plays an important role in the immune system independently of cancer progression by preventing autoimmune response as well as by regulating T cell development, differentiation and proliferation [57]. For instance, TGFβ mediates the differentiation of T helper (Th) cells into Th2 by repressing the transcriptional activity of T-bet and GATA3 [58]. Moreover, TGFβ can induce apoptosis in lymphocytes by activating the lipid phosphatase *SHIP* [59], [60] and can block dendritic cell (DC) maturation [61].

While the role of TGFβ secreted by tumour cells on the immune system has been widely studied, it is also important to consider that TGFβ can also be secreted by immune cells. In particular, tumour-associated macrophages (TAMs) and myeloid-derived suppressor cells (MDSC) secrete TGFβ into the tumour microenvironment [62]. Deletion of the type II TGFβ receptor in breast cancer cells leads to MDSCs infiltrating into the invasive front of the tumour, where they promote metastasis by producing TGFβ [63]. Hence, both tumour-derived or host immune-cell derived TGFβ can exert tumour-promoting roles acting on various immune cell populations.

### 3.1 Innate immune cells

TGFβ affects macrophages and their precursors, monocytes. TGFβ can also affect neutrophils the master regulators of inflammation and DCs, the professional antigen-presenting cells (Fig. 2). TGFβ stimulates monocyte migration [64] and promotes a de-activated or resting status in macrophages, resulting in a altered immune response [65]. Additionally, tumour-derived TGFβ can induce tumour-associated macrophage (TAM) polarization by suppressing nitric oxide [66], [67], [68], [69]. Tumour-derived TGFβ also promotes tumour-associated neutrophils (TANs) [70]. TANs are classified as N1 (anti-tumorigenic) and N2 (pro-tumorigenic) neutrophils; blocking TGFβ reduces N1 TAN infiltration, which in turn decreases activation of intra-tumoral CD8+T cells [70]. Finally, tumour-derived TGFβ induces DC apoptosis and inhibits DC migration in primary and secondary lymphoid organs as well as in metastatic tumour-draining lymph nodes [71], [72], [73].

### 3.2 Innate-like lymphocytes

NK cells are cytotoxic innate lymphoid cells (ILC) [74]. NK cytotoxicity is mediated by NK-specific receptors and co-receptors such as NKp46, NKp30, NKp44 and NKG2D, which serve as activating surface molecules [75]. TGFβ down-regulates NKp30 and NKG2D in human NK cells, thus inhibiting NK-mediated DC killing [76]. Similarly, in lung and colorectal cancers TGFβ plasma levels and NKG2D levels on NK cells are negatively correlated [77]. Since TGFβ downregulates activating surface molecules in NK cells, it can impair the recognition of tumour cells by NK cells and thus impede NK-mediated cytolysis and clearance of tumour cells (Fig. 2).

### 3.3 Adaptive immune cells

TGFβ secreted by cancer cells can also impact T cell activity by regulating their transcriptional profile. TGFβ directly targets cytotoxic T cells (CTLs) through transcriptional repression of cytotoxic genes, such as perforin, granzyme A, granzyme B, IFN-γ and FasL, resulting in tumour cell escape from immunosurveillance [78]. As a consequence, blockade of TGFβ signalling in T cells supports tumour-specific CD8+ cytotoxic T cells and promotes tumour eradication *in vivo* [79]. Moreover, knocking out TGFβ in mice or deleting SMAD family members in T cells result in altered T-cell homeostasis and thus promotes cancer initiation [80], [81], [82], [83].

One of the most important roles of TGFβ in promoting tumour escape from immunosurveillance is sustaining Tregs, which are mediators of self-tolerance [84] and support immunosuppression [85]. TGFβ induces FOXP3 expression and thus maintains CD4+CD25+FOXP3+ Tregs and their immunosuppressive functions through SMAD3 and NFAT mediated transcription [86], [87], [88]. Moreover, TGFβ secreted by lung cancer cells induces Treg cells in the lung tumour microenvironment [66]. In HCC, TGFβ has been reported to promote the differentiation of Tregs, whereas blockade of TGFβ decreases Tregs in liver tissues *in vivo*, thus reducing HCC progression [89]. In addition to Tregs, TGFβ also induces Th17 cells, which are involved in inflammation [90], [91] and it can inhibit IL-2-dependent T cell proliferation [92] (Fig. 2).

## 4 TGFβ signalling in cancer metastasis

### 4.1 TGFβ signalling in cancer cell motility and local invasion

**Fig. 3:**
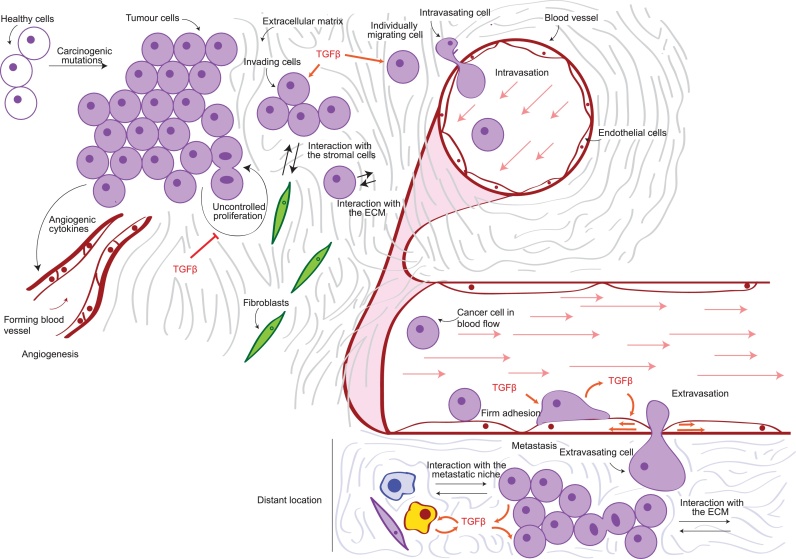
The metastatic cascade. Diagram summarising the progression of cancer metastasis. Proliferating cells begin invading through the extracellular matrix as groups or individually, interacting with other cells in the tumour microenvironment. They eventually encounter blood vessels, which they enter through a process known as intravasation. While in the vessel, cancer cells are transported by the blood flow all over the body. They eventually form loose attachments to the endothelial cells (tethering), which turn into firmer adhesion and eventually lead to extravasation, or exit from the vessel. The extravasated cells colonise the new metastatic niche by interacting with the extracellular matrix as well as with other cells in the tumour microenvironment and resuming proliferation to form a secondary tumour. TGFβ effects are highlighted in orange.

Metastasis is the spreading of cancer cells throughout the body and is the main cause of cancer-related deaths [93]. It is a multi-step process where cancer cells leave the primary tumour, disseminate to distant sites and form secondary tumours [94] (Fig. 3). During the initial stages of metastasis, tumour cells lose cell–cell contacts and acquire migratory abilities, invading the local tumour microenvironment. During Epithelial to Mesenchymal Transition (EMT), expression of epithelial cell–cell adhesion proteins such as E-cadherin, ZO-1 and occludin is down-regulated, while mesenchymal proteins like N-cadherin are up-regulated. This switch in gene expression is regulated by the Snail/Slug, ZEB1/2 and Twist transcription factors [1], [5], [38]. EMT not only induces “mesenchymal” motile characteristics in cancer cells, but also supports tumour initiation, host immunosurveillance evasion and chemoresistance [5] (Fig. 3).

TGFβ is a key driver of EMT in epithelial cancers [12], [95], [96]. In lung cancer, TGFβ-driven transcription regulates E-cadherin [97], Snail [98], N-cadherin [99] and vimentin [99], [100], [101], [102], [103], [104]. Similarly, TGFβ induces EMT in breast cancer cells, where it induces the expression of Sox4, thus promoting mesenchymal programmes, tumour progression and invasiveness [105]. TGFβ signalling also induces AP1 expression, which in turn regulates various mesenchymal and invasion-associated genes [106]. Importantly, TGFβ-induced Snail or Twist1 can in turn drive epigenetic changes that influence EMT [105]. Moreover, TGFβ regulates gene expression of integrins both in lung and breast cancer, resulting in increased cell motility, dissemination and metastasis [98], [107], [108], [109]. In HCC, EMT driven by TGFβ promotes cell dissemination and intrahepatic metastasis, in collaboration with other signalling pathways. TGFβ promotes EMT by inducing SNAIL1, conferring resistance to apoptosis [110]. Additionally, autocrine TGFβ promotes CXCR4 expression in HCC cells, driving cell migration and invasion [111], while TGFβ secreted from tumour associated macrophages (TAMs) induces cancer stem cell properties in HCC [112]. Furthermore, in prostate cancer TGFβ represses E-cadherin and promotes the expression of N-cadherin, ZEB1, TWIST, fibronectin and SNAIL1 [113], [114], [115]. TGFβ also supports EMT in prostate cancer cells by regulating NEDD9 [116].

In addition to epithelial cancers, TGFβ signalling also drives cell motility and local invasion in non-epithelial cancers. Glioblastoma, a grade IV malignant glioma that arises from glial cells, is one of the most common and aggressive brain tumours and it is characterised by its ability to infiltrate adjacent healthy brain [117], [118]. Glioblastomas are highly heterogeneous and can be classified into different sub-types, namely mesenchymal, classical, neural and pre-neural. In particular, mesenchymal glioblastoma presents with the highest correlation with EMT-related genes [119]. TGFβ has been shown to activate EMT drivers ZEB1 and SNAIL1 in glioblastoma, thus promoting motility and local invasion [120], [121]. Furthermore, TGFβ drives the expression of LIF through SMAD-mediated transcription in glioma-initiating cells [122]. LIF activates JAK/STAT signalling, promoting glioma cell self-renewal [122]. Moreover, TGFβ promotes glioblastoma cell motility by transcriptionally activating surface molecules such as cadherin-11 [36] and integrins [123], which can feed back to TGFβ-driven transcription by affecting SMAD2 activation [124].

Mesenchymal tumours switch between different modes of individual migration [125]. In particular, melanoma cells switch between rounded-amoeboid motility, driven by actomyosin contractility, and elongated-mesenchymal motility, dependent on higher levels of Rac dependent adhesion [126]. TGFβ-SMAD2-CITED1-mediated transcription promotes melanoma amoeboid invasion [127]. Specifically, TGFβ-SMAD2-CITED1 regulate expression of LIF and JAK1 [47], [127] and of the RhoGEF ARHGEF5 [128], both of which support actomyosin contractility [127], [129]. TGFβ signalling also favours detachment of melanoma cells from keratinocytes [127], which is necessary for melanomas to escape the epithelial niche and invade into the dermal layers. Perhaps as a consequence of its role in regulating amoeboid motility, TGFβ-driven transcription has been widely recognised as a promoter of invasion in melanoma [130], [131], [132], [133], [134], [135]. Since lung cancer has also been described to engage in amoeboid invasive strategies [136], it will be important to assess if TGFβ controls this particular invasive behaviour in lung cancer cells.

### 4.2 TGFβ signalling in crossing the endothelial barrier

Following local invasion, cancer cells enter blood or lymphatic vessels in a process known as intravasation [137]. The blood flow subsequently transports cancer cells throughout the body, until they exit the vasculature and form secondary tumours [138] (Fig. 3). In breast cancer, TGFβ-induced EMT activates CCR7/CCL21-mediated chemotaxis, which promotes targeted migration through lymphatic vessels [139]. While the role of TGFβ in intravasation remains unclear, it has been suggested that TGFβ-driven transcription is able to regulate cancer cell extravasation in lung, breast cancer and HCC cells [140], [141], [142]. Moreover, in melanoma TGFβ-driven transcription promotes adhesion to endothelial cells [127], as well as extravasation [130], [141]. On the other hand, TGFβ also favours cell extravasation by acting directly on the endothelium. For example, TGFβ activates transcription of α-smooth muscle actin (SMA) in endothelial cells favouring melanoma cells extravasation [141]. Nevertheless, more work is needed to fully understand the role of TGFβ in regulating endothelial homeostasis during cancer dissemination.

### 4.3 TGFβ signalling in secondary organ colonisation

Cancer cells that reach a secondary site after extravasation need to proliferate to form secondary tumours (Fig. 3). In lung cancer, TGFβ has been shown to support metastasis in mouse models [143]. In fact, activation of TGFβ-dependent transcription by R-SMAD activators, such as profilin2 and PREP1, results in enhanced metastasis formation [29], [104]. Moreover, TGFβ/Snail-driven EMT suppresses fatty acid synthase (FASN) expression in lung cancer cells, which is sufficient to stimulate migration and extravasation *in vitro*, as well as lung metastasis *in vivo* [144]. In breast cancer, TGFβ induces HMGA2 expression via SMAD signalling during EMT [105], which induces metastasis [145]. Furthermore, loss-of-function mutations in TGFβ repressors such as MED12 [146], SIRT1 [147] and DEAR1 [148] results in invasion and metastasis. In HCC, TGFβ induces long non-coding RNA LncRNA-ATB, which activates the invasion-metastasis cascade [149]. LncRNA-ATB [150] and LncRNA-HIT [151] high expression levels have also been associated with EMT, invasion and metastasis in breast cancer. In addition, TGFβ-induced lysyl oxidase-like 2 (LOXL2) transcription may also contribute to HCC intrahepatic and extrahepatic metastasis by modifying the tumour microenvironment and metastatic niche [152]. In prostate cancer, TGFβ-driven transcription has been linked to bone metastasis through the activation of mTOR pathway [153], [154], [155] and TGFβ-dependent ALCAM expression drives bone metastasis [155]. In melanoma, TGFβ-SMAD2-CITED1 mediated transcription is necessary for melanoma metastasis [127]. Moreover, TGFβ derived from platelets promotes melanoma metastasis formation [156] and expression of EWI2 – a negative regulator of TGFβ signalling – is associated with decreased metastasis formation [157].

## 5 Concluding remarks

TGFβ-induced transcription exerts a profound influence on tumour cells and stroma. Strong evidence indicates that while early in cancer progression TGFβ plays a tumour suppressor role, in later stages it is a potent pro-metastatic mediator. TGFβ can therefore be considered a general metastasis promoter and an interesting therapeutic target.

Several inhibitors of the TGFβ pathway are being developed and clinically tested for a number of cancers, including glioma, pancreatic cancer, non-small-cell lung carcinoma, advanced HCC and melanoma [158], [159], [160], [161]. A phase II clinical trial with Galunisertib, a TβRI inhibitor, is currently on-going in patients with advanced HCC (NCT01246986, http://clinicaltrials.gov). Moreover, a vaccine targeting TGFβ2 (belagenpumatucel-L) has undergone phase III clinical trials in lung cancer patients [162], where it has yielded promising results. In metastatic melanoma patients, the two most promising drugs targeting TGFβ signalling are Fresoluminab (GC1008, Genzyme) and Trabedersen (AP-12009, Antisense Pharma), both targeting the TGFβ ligands. GC1008 has been tested in phase I/II trials, where it has obtained mixed results probably reflecting the contrasting roles of TGFβ. GC1008 hindered metastatic progression of melanoma, but also lead to the development of non-melanoma cutaneous malignancies [163].

These past and current trials aimed to target either TGFβ ligands or their receptors. Therefore, they are subject to dangerous side-effects and reduced effectiveness as a result of their impact on the tumour suppressing actions of TGFβ. However, the body of work presented in this review clearly indicates that the transcriptional effects of TGFβ signalling are key to mediate its pro-metastatic effects. Thus, we can hypothesize that drugs directed against the transcriptional targets and regulators of the TGFβ pathway might be able to block the pro-metastatic effects of TGFβ signalling without compromising its tumour suppressor role.

Importantly, the tumour microenvironment should be taken into consideration when targeting the TGFβ pathway. Considering the discussed effects TGFβ exerts on both innate and adaptive immune cells, it is essential to understand how targeting the TGFβ pathway would affect tumour immunity. Immune cell screening, such as Treg frequency and phenotypic alterations, systemically and in the tumour site before and after TGFβ-targeted therapy could be incorporated as potential prognostic tools for cancer patients.

## Conflict of interest statement

The authors declare that there are no conflicts of interest.

## Funding source

Cancer Research UK (CRUK) C33043/A12065 (VSM and ECM).

Royal Society RG110591 (VSM).

Medical Research Council C97993H (GC).

NIHR BRC at Guy’s & St. Thomas’ NHS Foundation Trust and KCL Ph.D. Programme in Biomedical and Translational Science (MG).
